# Draft Genome Sequence of *Cryobacterium roopkundensis* Strain RuGl7, Isolated from a Soil Sample in the Vicinity of Roopkund Lake, Himalayas, India

**DOI:** 10.1128/genomeA.01206-14

**Published:** 2014-11-20

**Authors:** Gundlapally Sathyanarayana Reddy, Ara Sreenivas, Sisinthy Shivaji

**Affiliations:** CSIR-Centre for Cellular and Molecular Biology, Habsiguda, Hyderabad, Telangana, India

## Abstract

We report the 4.36-Mb genome of *Cryobacterium roopkundensis* strain RuGl7, isolated from a soil sample collected in the periphery of Roopkund Lake, Himalayas, India. The draft genome consists of 4,356,863 bp, 4,048 protein-coding sequences, and 50 RNAs, with 65.3% G+C DNA content.

## GENOME ANNOUNCEMENT

*Cryobacterium roopkundensis* strain RuGl7 was isolated from a soil sample collected at the periphery of the Roopkund glacial lake, Himalayas, India (1). The bacterium is Gram positive, aerobic, rod-shaped, and motile, grew between 15 and 18°C, and is phylogentically related to the genera *Rathayibacter* and *Clavibacter*. This is the first species to be sequenced from the genus *Cryobacterium.*

The genome was sequenced using the Roche 454 (FLX Titanium) pyrosequencing platform. The sequencing yielded a genome size of 48,225,101 bases in 139,076 reads, with 11× coverage. The sequences were assembled using GS De Novo Assembler (version 2.8; F. Hoffmann-La Roche Ltd., Switzerland) and it yielded 226 contigs (4,356,863 bp) with a G+C DNA content of 65.3%. The average contig size was of 19,278 bp and the largest contig was 132,366 bp in length. Genome annotation was performed using Rapid Annotation using Subsystem Technology (RAST) (2), and tRNA and rRNA genes were predicted by tRNAscan-SE (v 1.23) (3) and RNAmmer (version 1.2) (4), respectively.

The annotation detected 4,048 coding sequences (including 1 each for 5S rRNA, 23S rRNA, and 16S rRNA) and 47 tRNA genes. The annotated genome contains 33 genes encoding resistance against antibiotics and toxic compounds; 1 each for resistance to mercury, streptothricin, and chromium; 3, 4, 4, and 5 genes for resistance to cobalt-zinc-cadmium, β-lactamase, fluoroquinolones and arsenic, respectively; and 14 genes for copper homeostasis. There are 123 genes for membrane transport, including 54 ABC transporters, 17 protein-nucleoprotein secretion systems, and 21 cation transporters. Among 117 predicted stress response genes, 3 belong to the *cspA* family of genes involved in cold stress. There are 27 genes responsible for nitrogen metabolism. The genome comprises 12, 42, 47, and 178 genes involved in potassium, sulfur, phosphorus, and protein metabolism, respectively, of which serine endopeptidase (EC 3.4.21) complements the cleavage of gelatin at low temperatures by strain RuGl7 (1). Interestingly, strain RuGl7 encompasses 5 genes involved in photosynthesis, mainly related to proteorhodopsin. The whole-genome sequencing of *Cryobacterium roopkundensis* will accelerate research on cold adaptation of psychrophilic microorganisms.

### Nucleotide sequence accession number.

The draft genome sequence of *Cryobacterium roopkundensis* RuGl7 has been deposited at DDBJ/EMBL/GenBank under the accession no. JPXF00000000. The version described in this paper is the first version.
